# A Treatise on Hernia: Comprising the Surgical Anatomy, Operative Surgery, and Treatment

**Published:** 1838-07

**Authors:** 


Art. XIII.—*A Treatise on Hernia: comprising the Surgical Anatomy, Operative Surgery, and Treatment.* By M. W. Hilles.—*London,* 1838. 12mo. pp. 52.

We regard this little treatise as a correct epitome of the Anatomy and Operative Surgery of Hernia, and as likely to be useful. So much is here condensed into a narrow compass, than we can scarcely recollect anything of anatomical interest or practical nature connected with the subject, that Mr. Hilles has not culled, and interwoven in his pages. Nothing original is, we presume, affected by our author, if we except a reference to what is styled "a newly proposed operation for strangulated hernia," and which consists merely in applying the taxis to the sac after it is laid bare, without opening into it, and to which we formerly adverted, (Vol. IV. p. 256.) To compile, however, with brevity and yet with judgment is in itself a merit, and entitles our author to the attention of the surgical student; who, so far as hernia is concerned, will find in this little pamphlet a useful companion in the dissecting room, both in refreshing his memory, and adding to its store.

